# 1718. Effectiveness of 13-valent pneumococcal conjugate vaccine for prevention of invasive pneumococcal disease among children in the United States between 2010 and 2019: an indirect cohort study

**DOI:** 10.1093/ofid/ofad500.1550

**Published:** 2023-11-27

**Authors:** Kristin Andrejko, Ryan Gierke, Jemma Rowlands, Jennifer Rosen, Ann Thomas, Zachary Landis, Maria Rosales, Susan Petit, William Schaffner, Corinne Holtzman, Monica M Farley, Meghan Barnes, Lee Harrison, Lesley McGee, Sopio Chochua, Jennifer Verani, Adam Cohen, Tamara Pilishvili, Miwako Kobyashi

**Affiliations:** CDC; Centers of Disease Control and Prevention, Atlanta, Georgia; New York State Department of Health, Albany, New York; NYC Department of Health and Mental Hygiene, Long Island City, New York; Oregon Health Authority, Portland, Oregon; Emerging Infections Program University of New Mexico, Albuquerque, NewMexico; California Emerging Infections Program, Oakland, California; Connecticut Department of Public Health, Hartford, Connecticut; Vanderbilt University Medical Center, Nashville, Tennessee; Minnesota Department of Health, St. Paul, Minnesota; Emory University School of Medicine, Division of Infectious Diseases, Atlanta, Georgia; Colorado Department of Public Health and Enviroment, Denver, Colorado; University of Pittsburgh, Pittsburgh, Pennsylvania; Centers for Disease Control and Prevention, Atlanta, Georgia; CDC; Centers for Disease Control, Atlanta, Georgia; CDC; Centers for Disease Control and Prevention, Atlanta, Georgia; CDC

## Abstract

**Background:**

The 13-valent pneumococcal conjugate vaccine (PCV13) was introduced for infant use in the United States in 2010, replacing PCV7. A U.S. case-control study (2010–2014) demonstrated vaccine effectiveness (VE) for ≥1 dose of PCV13 at 86%; however, this study lacked statistical power to examine VE by number of doses and against individual serotypes.

**Methods:**

We used the indirect cohort method to estimate VE of PCV13 against vaccine-type invasive pneumococcal disease among children < 5 years in the U.S. from May 1, 2010 to December 31, 2019. We included IPD cases identified through CDC’s Active Bacterial Core surveillance in 10 U.S. states; during 2010 – 2014, we additionally included cases enrolled in a post-licensure matched case-control study from expanded sites. Cases and controls were defined as individuals with PCV13-type-IPD (VT-IPD) and non-PCV13-type-IPD (NVT), respectively; serotype 6C was categorized as VT. We used logistic regression to estimate the adjusted odds of prior PCV13 receipt, controlling for confounders identified a priori including age category, race/ethnicity, sex, state, year, and any immunocompromising and/or chronic conditions.

**Results:**

A total of 1,161 IPD cases were identified; 223 (19.2%) were VT cases and 938 (80.8%) were non-VT controls. Of those, 108 cases (48.4%; 108/223) and 600 controls (64.0%; 600/938) had received >3 PCV13 doses; 47 cases (21.1%) and 53 controls (5.7%) had received no PCV doses. Serotypes 19A (N=96), 3 (N=60), and 19F (N=45) caused 90.1% (201/223) of VT-IPD. VE of >1 or ≥3 PCV13 doses against VT-IPD was 81.7% (95% Confidence Interval: 69.1–89.1%) and 87.8% (75.2–94.0%), respectively. VE of ≥3 PCV13 doses was 87.0% (75.8–93.0%), 54.6% (-8.8–81.0%), and 92.9% (74.4–98.0%) against serotypes 19A, 3, and 19F, respectively. VE was 87.6% (67.9-95.2%) for three primary doses before 12 months of age and 92.4% (78.2–97.2%) for three primary doses and a booster at 12 months of age or older.

Vaccine effectiveness estimates against PCV13-type IPD among US children under five years of age, 2010 - 2019

**Figure:**
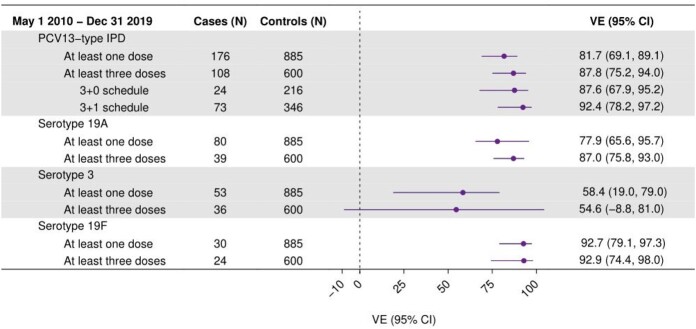

**Conclusion:**

VE of ≥1 PCV13 dose against IPD was consistent with the previous estimate from the case-control study, and ≥3 doses appear to provide substantial protection. Among the most commonly circulating VT-IPD, PCV13 was protective against serotypes 19A and 19F IPD; protection against serotype 3 IPD did not reach statistical significance.

**Disclosures:**

**Lee Harrison, MD**, GSK: Advisor/Consultant|Merck: Advisor/Consultant|Pfizer: Advisor/Consultant|Sanofi: Advisor/Consultant **Tamara Pilishvili, PhD MPH**, GSK: Employed by GSK since February 2023. At the time of data collection and analysis for this work was employed by the CDC

